# Effect of soluble dietary fiber extracted from *Lentinula edodes* (Berk.) Pegler on lipid metabolism and liver protection in mice on high-fat diet

**DOI:** 10.3389/fnut.2025.1537569

**Published:** 2025-01-30

**Authors:** Kangxiao Guo, Jing Liu, Zihan Yao, Zhoujin Tan, Tao Yang

**Affiliations:** ^1^National Engineering Laboratory for Rice and By-Product Deep Processing, College of Food Science and Engineering, Central South University of Forestry and Technology, Changsha, China; ^2^Department of Pharmacy, Changsha Health Vocational College, Changsha, China; ^3^College of Chinese Medicine, Hunan University of Chinese Medicine, Changsha, China

**Keywords:** *Lentinusedodes*, soluble dietary fiber, lipid metabolism, liver protection, high fat diet

## Abstract

With the increasing annual production of *Lentinula edodes*, the residues of *Lentinus edodes* are mass produced and wasted every year. In order to further explore the added value and effective utilization of *Lentinus edodes*, we studied the lipid-lowering efficacy and liver protective effect of *Lentinus edodes* soluble dietary fiber in mice on high-fat diet. Project team from *Lentinus edodes* extracted soluble dietary fiber, and its physicochemical properties, selected 30 male mice, randomly divided into normal group (N), high fat diet group (F), add low dose dietary fiber high fat diet (FL), add medium dose dietary fiber high fat diet group (FM), add high dose dietary fiber high fat diet group (FH) five groups. After 4 weeks, we assessed general state, organ conditions, liver status, blood parameters, expression of hepatic lipid metabolism genes, mRNA levels of key hepatic lipid metabolism genes. The results showed that the molecular weight of soluble dietary fiber is about 17.029 kDa, and the monosaccharides such as galactose, glucose and mannitol are connected by β-glycosidic bond. The soluble dietary fiber of *Lentinus edodes* can effectively slow the weight growth due to high-fat diet, delay liver tissue lesions, reduce the levels of ALT, AST, ACP, LDL-C, TG, TV, FFA, SOD, GSH and MDA, and increase the levels of γ-GT, HDL-C and CAT in blood. *Lentinus edodes* soluble dietary fiber decreased the expression of AMPKα and SREBP-2 in the liver, and increased the expression of PPARα, ACS, CPT1a, CYP7A1. It is proved that the soluble dietary fiber of *Lentinus edodes* can alleviate the organ fat accumulation caused by high-fat diet to some extent, effectively combat the liver injury, oxidative stress pressure and lipid metabolism disorder caused by high-fat diet, and provide an experimental basis for the subsequent effective use of soluble dietary fiber of *Lentinus edodes* in fat reduction.

## 1 Introduction

*Lentinula edodes* (Berk) Pegler (*Lentinus edodes*) is the second largest edible fungus in the world. Because *Lentinus edodes* taste delicious, unique aroma and rich nutrition, they are deeply loved by the majority of consumers. *Lentinus edodes* are not only rich in dietary fiber, protein and minerals, but also rich in *Lentinus edodes* polysaccharides, *Lentinus* purines*, Lentinus edodes* peptides and other active substances. It has high nutritional, medicinal and health value, and belongs to the food and drug homologous food (1). *Lentinus edodes* is considered to be a good alternative source of dietary fiber (2). Compared with insoluble dietary fiber (IDF), soluble dietary fiber (SDF) is more popular because it can alleviate hyperlipidemia, diabetes, cardiovascular diseases, colon cancer and other diseases (3). However, with the continuous increase of the annual production of *Lentinula edodes*, the residues of *Lentinula edodes* are produced and wasted every year. Therefore, the added value of *Lentinus edodes* needs further exploration and effective utilization.

In recent years, with the continuous improvement of people’s living standards, the intake of meat, dairy products, fat, sugar and other high-energy foods has increased, leading to the rising prevalence of overweight and obesity (4, 5). At present, obesity has become one of the world’s public health problems. Obesity is caused by excessive fat accumulation in the body, which is usually caused by excessive food intake or changes in body metabolism, resulting in excessive weight gain and pathophysiological process. Obesity not only brings inconvenience to our life and work, but also is an important risk factor for metabolic diseases such as hypertension, hyperlipidemia, cardiovascular and cerebrovascular diseases (6). In clinical practice, we mainly use the means of strengthening physical exercise and drug treatment, but the effect is not obvious, and there are some side effects. Dietary intervention has the advantage of no side effects in the prevention and treatment of obesity, which is of great significance for the treatment of obesity (7). Dietary fiber diet is a dietary adjustment auxiliary program gradually applied to clinical obese patients in recent years. Studies have shown that dietary fiber can promote gastrointestinal peristalsis, facilitate food digestion and absorption, and prevent and inhibit the occurrence of obesity, diabetes and other diseases (8). Compared with oat dietary fiber based on β-glucan, the dietary fiber in *Lentinus edodes* includes cellulose, hemicellulose and some polysaccharides, which may have more abundant functions, and its efficacy may be related to intestinal health (9, 10). Both *Lentinus edodes* soluble dietary fiber and psyllium dietary fiber can promote intestinal health, but the specific effects may be different. The active substances in *Lentinus edodes* dietary fiber are believed to directly or indirectly affect the immune system, while psyllium acts on the urinary system. Because of psyllium’s special taste and properties, it may be more used in the production of specific foods (11).

In this study, soluble dietary fiber was extracted from *Lentinus edodes,* and the physicochemical properties of the extracted soluble dietary fiber were preliminarily analyzed. By adding different concentrations of *Lentinus edodes* soluble dietary fiber to mice on a daily high-fat diet, and observing the general state of the mice, the effects of *Lentinus edodes* soluble dietary fiber on liver protection, liver lipid metabolism, liver oxidative stress response and liver to key lipid metabolism genes expression in mice on a high-fat diet were investigated, and the lipid-lowering efficacy and liver protection of *Lentinus edodes* soluble dietary fiber were evaluated.

## 2 Materials and methods

### 2.1 Extraction of soluble dietary fiber from *Lentinus edodes*

Soluble dietary fiber of *Lentinus edodes* was according to Wu Liping’s method (12).

### 2.2 Preliminary identification of the properties of soluble dietary fiber in *Lentinus edodes*

#### 2.2.1 Identification of basic physicochemical properties of soluble dietary fiber extracted from *Lentinus edodes*

The soluble dietary fiber of *Lentinus edodes* extracted from 20.00 mg was accurately weighed, dissolved in ultra-pure water and contained in a 20 mL volumetric flask to obtain 1.0 mg/mL soluble dietary fiber solution of *Lentinus edodes.* Molicit reaction (Molish), starch detection, ninhydrin reaction and sulfate-carbazole reaction were performed. The specific method is shown in Table 1.

**Table 1:** Basic physicochemical properties of soluble dietary fiber in *Lentinus edodes.*

No.	Name of experiment	Experimental method	Experimental phenomena to be observed	Reference
1	Molish reaction	1 mL of *Lentinus edodes* soluble dietary fiber solution was put into a clean test tube, and 2 drops of 10% α-naphthol ethanol solution were added into the test tube. After the solution was mixed evenly, 1 mL of concentrated sulfuric acid was slowly added along the tube wall, and the color of the connection between the two liquid surfaces was observed.	If there is a purple red ring in the solution, it indicates that the sample contains sugar compounds.	(58)
2	Starch detection	1 mL of *Lentinus edodes* soluble dietary fiber solution was put into a clean test tube, and iodine solution was added to the test tube to observe whether the sample in the test tube was turned blue.	If the solution was turned blue, the sample contains starch.	(59)
3	Triketohydrindene reaction	1 mL of *Lentinus edodes* soluble dietary fiber solution was put into a clean test tube, and 0.5 0.1% ninhydrin ethanol solution was added to the test tube. Shake the solution well, boil it, cool it for 5 min, and observe whether the solution in the test tube has color change. The positive control was 0.5% amino acid and the negative control was distilled water.	If a blue purple substance is generated in the solution, it means that the sample contains amino acids; If brown substance is generated in the solution, it indicates that aspartic acid is contained in the sample; If the yellow substance in the solution is generated, it indicates that the sample contains proline or hydroxyproline.	(60)
4	Ulfate-carbazole reaction	1 mL of *Lentinus edodes* soluble dietary fiber solution was put into a clean test tube, the test tube was put into an ice water bath, then 5.0 mL of concentrated sulfuric acid was slowly added to the test tube, cooled to room temperature, 0.2 mL of 0.15% carbazole anhydrous ethanol solution was added to the test tube, shaken in a boiling water bath for 15 min, and the color change of the solution in the test tube was observed.	If purple is produced in the solution, it indicates that the sample contains uronic acid.	(61)

#### 2.2.2 Molecular weight distribution determination of soluble dietary fiber extracted from *Lentinus edodes*

The samples were dissolved in 0.1 M NaNO_3_ aqueous solution containing 0.02% NaN_3_ at the concentration of 1 mg/mL and filtered through a filter of 0.45 μm pore size. The homogeneity and molecular weight of various fractions were measured using SEC-MALLS-RI. The weight and number-average molecular weight (Mw and Mn) and polydispersity index (Mw/Mn) of various fractions in 0.1 M NaNO_3_ aqueous solution containing 0.02% NaN_3_ were measured on a DAWN HELEOS-II laser photometer (Wyatt Technology Co., USA) equipped with two tandem columns (300 × 8 mm, Shodex OH-pak SB-805 and 803, Showa Denko K.K., Tokyo, Japan) which was held at 45°C using a model column heater by Sanshu Biotech. Co., LTD (Shanghai, China). The flow rate is 0.6 mL/min. A differential refractive index detector (Optilab T-rEX, Wyatt Technology Co., USA) was simultaneously connected to give the concentration of fractions and the dn/dc value. The dn/dc value of the fractions in 0.1 M NaNO_3_ aqueous solution containing 0.02% NaN_3_ was determined to be 0.141 mL/g (13).

#### 2.2.3 Determination of the extracted soluble dietary fiber monosaccharide composition of *Lentinus edodes*

Approximately 5 mg of sample was hydrolyzed with trifluoroacetic acid (2 M) at 121°C for 2 h in a sealed tube. Dry the sample with nitrogen. Add methanol to wash, then blow dry, repeat methanol wash 2–3 times. The residue was re-dissolved in deionized water and filtered through 0.22 μm microporous filtering film for measurement. The sample extracts were analyzed by high-performance anion-exchange chromatography (HPAEC) on a CarboPac PA-20 anion-exchange column (3 by 150 mm; Dionex) using a pulsed amperometric detector (PAD; Dionex ICS 5000+ system). Flow rate, 0.5 mL/min; injection volume, 5 μL; solvent system A: (ddH_2_O), solvent system B: (0.1 M NaOH), solvent system C: (0.1 M NaOH, 0.2 M NaAc); gradient program, volume ratio of solution A, B, C was 95:5:0 at 0 min, 85:5:10 at 26 min, 85:5:10 at 42 min, 60:0:40 at 42.1 min, 60:40:0 at 52 min, 95:5:0 at 52.1 min, 95:5:0 at 60 min (14).

#### 2.2.4 Electron microscopic scanning analysis of soluble dietary fiber extracted from *Lentinus edodes*

The molecular morphologies of polysaccharides were observed using a scanning electronic microscope (Zeiss Merlin Compact, Germany). The samples, coated with a thin gold layer, were placed on the sub-strate, and the images were then observed at a voltage of 1.0 kV with 500- and 10,000-fold magnification under high vacuum by Sanshu Biotech. Co., LTD (Shanghai, China) (15).

#### 2.2.5 Infrared scanning of soluble dietary fiber from *Lentinus edodes*

Fourier Transform infrared (FT-IR) spectra of polysaccharides were determined using a spectrometer (Nicolet iZ-10, Thermo Nicolet, USA). The polysaccharide samples were mixed with KBr powder and then pressed into 1 mm pellets for FT-IR measurement in the range of 4,000 to 400 cm^−1^ by Sanshu Biotech. Co., LTD (Shanghai, China) (16).

### 2.3 Animals and diet

30 male SPF-grade KM mice (4 weeks old, body weight 20 ± 2 g, No. SCXK2019-0004) were obtained from Hunan Slake Kingda Laboratory Animal Co.

All animal experiments were conducted following the guidelines of the Experimental Animal Ethics Committee of Hunan University of Traditional Chinese Medicine (approval number: LL2023032901). Mice were housed in an environmentally controlled room maintained at a temperature of 20 ± 2°C, humidity of 40–60%, and a light–dark cycle of 12 h. After acclimatization to one week of feeding under specific pathogen-free conditions, the mice were randomly divided into 5 treatment groups (*n* = 6). The first group (N) was fed a normal diet (23.07% protein, 11.85% fat, 65.08 carbohydrates). The second group (F) was fed a high-fat diet (basal diet with 15 percent lard, 20 percent sucrose, 1.2 percent cholesterol, and 0.2 percent sodium cholate). The third group (FL) was fed high-fat chow, and 150 mg/kg of water-soluble dietary fiber from *Lentinus edodes*. The fourth group (FM) was fed high-fat chow, and 300 mg/kg of water-soluble dietary fiber from *Lentinus edodes*. The fifth group (FH) was fed high-fat chow, and 450 mg/kg of water-soluble dietary fiber from *Lentinus edodes*.

Euthanasia of mice was typically performed using methods that ensure minimal pain and distress. We used the carbon dioxide (CO_2_) inhalation method, where mice were exposed to a gradual fill of CO_2_ in a chamber to induce unconsciousness and death. This method was often followed by a secondary physical method to confirm death, such as cervical dislocation or decapitation. Injectable anesthetics like pentobarbital were also be administered to induce a deep state of anesthesia before death. All methods comply with ethical guidelines and regulations set by institutional and governmental bodies to ensure humane treatment.

### 2.4 Histological observations

Freshly collected liver tissues from all groups of mice were sliced and soaked in 10% formalin overnight, then gradient dehydrated and embedded in paraffin wax, after which the liver tissue blocks were cut into 6 μm thick sections and stained with hematoxylin and eosin (H&E) staining for morphological observation. The liver condition was evaluated by the balloon like change in NASH-CRN scoring standard (17).

### 2.5 Organ coefficient studies

Spleen and thymus tissues were freshly collected from each group of mice, weighed, and the corresponding organ index was calculated using the formula (18):


$$\mathrm{Spleen index}=\mathrm{spleen weight}\;\left(g\right)/\mathrm{mouse body weight}\;\left(g\right)\times 100\%$$

$$\begin{array}{l} \mathrm{Thymus index}=\mathrm{weight of thymus}\;\left(g\right)/\mathrm{body weight of}\\ \;\mathrm{mice}\;\left(g\right)\times 100\% \end{array}$$

### 2.6 Biochemical analysis and cytokine measurement

The serum concentrations of triglyceride (TG), total cholesterol (TC), low-density lipoprotein cholesterol (LDL-C), high-density lipoprotein cholesterol (HDL-C), Serum total bile acids (TBA), Alanine aminotransferase (ALT), Gamma-glutamyltransferase (γ-GT), Aspartate aminotransferase (AST), Acid phosphatase (ACP) contents of malondialdehyde (MDA), catalase (CAT), superoxide dismutase (SOD), and glutathione peroxidase (GSH-Px) in liver were analyzed using commercial kits (Konodi Bio Ltd.)

### 2.7 Effect of *Lentinus edodes* soluble dietary fiber on the expression of genes related to liver lipid metabolism in mice fed with high fat diet

Fresh liver tissue samples of each experimental group were taken, and immediately frozen with liquid nitrogen. After the tissue sample was ground into fine powder with a precooled mortar and pestle, 100 mg of fine powder was quickly weighed. The RNA in the sample was extracted in strict accordance with the instructions of the universal RNA extraction kit. After the 5 μL RNA sample was diluted 10 times with ddH_2_O, the OD260 value of RNA was determined. After the concentration of RNA in the sample was unified, the RNA sample was reverse transcribed into cDNA in strict accordance with the instructions of the Takara reverse transcription kit, and stored at −20°C (19).

0.5 μL of upstream and downstream primers, 2.0 μL of cDNA sample and 2.0 μL of ddH_2_O were added into the octuple, respectively. The solution was mixed and centrifuged at low speed for 30 s. 5 μL of SYBR enzyme was added into the octuple under dark conditions. The solution was mixed and centrifuged at low speed for 30 s. With GADPH as the internal reference, AMPKα, SREBP2, PPARα, CPT1A, ACS and CYP7A1 were determined by fluorescence quantitative PCR. The CQ value was automatically calculated by the system, and the relative gene expression was calculated by 2^−ΔΔ^ method. The base sequence and product size of the mouse derived gene primers used are shown in Table 2.

**Table 2:** Primer base sequence and product sizes of the source gene.

Number	Gene	Base sequence	Product size
1	GADPH	F: AGGAGCGAGACCCCACTAACA R: AGGGGGGCTAAGCAGTTGGT	247 bp
2	AMPKα	F: GGACTTACTTGTTGGATTTCCG R: CCTTTGGCAAGATCGATAGTTG	113 bp
3	SREBP-2	F: TTTTACTGAAGTAGAGCGGGTC R: CATGCATGGCTCTACAGGTATA	92 bp
4	PPARα	F: GAGCTGCAAGATTCAGAAGAAG R: GAATCTTTCAGGTCGTGTTCAC	171 bp
5	CPT1a	F: TCAAGAATGGCATCATCACTGG R: CGATGTTCTTCGTCTGGCTTG	177 bp
6	ACS	F: ATGTACGATGGCTTCCAGAGGG R: GGGACGACCACCATTGAGTAAGA	254 bp
7	CYP7A1	F: GTGATGTTTGAAGCCGGATATC R: TTTATGTGCGGTATTGAACAAG	168 bp

### 2.8 Data analysis

Chromatographic data were processed using the software ASTRA6.1. The CQ value of gene expression related data was automatically calculated by the built-in software, and the relative gene expression was calculated by 2^−ΔΔ^ method. All data were presented as mean ± standard deviation, and analyzed by SPSS 22.0 software. Independent sample T-test was used to analyze the differences between the two groups. *p* < 0.05 indicates a significant difference between the two groups, and *p* < 0.01 indicates a very significant difference between the two groups.

## 3 Results

### 3.1 Extraction of soluble dietary fiber from *Lentinus edodes*

**Figure 1:**
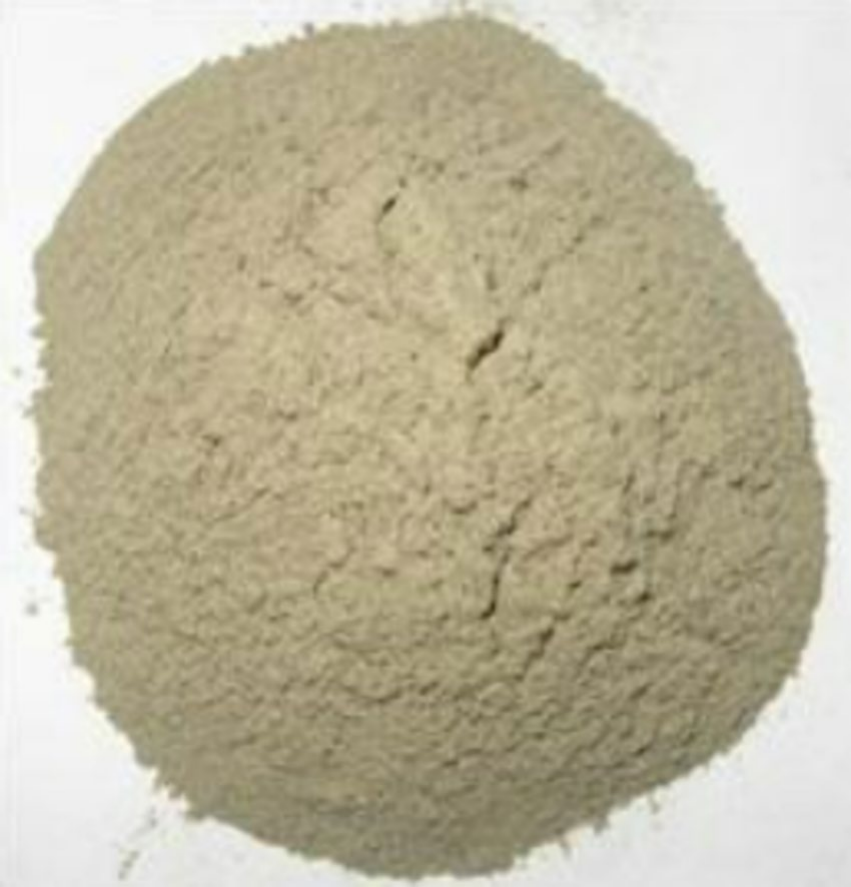
Extracted soluble dietary fiber from *Lentinus edodes.*

The extracted *Lentinus edodes* soluble dietary fiber was a light yellow powder with a soft texture and small particles (Figure 1). Preliminary physicochemical analysis of the extracted *Lentinus edodes* soluble dietary fiber showed that the extracted *Lentinus edodes* soluble dietary fiber contained saccharides and alditric acid, whereas there was no starch and amino acid (Table 3).

**Table 3:** Preliminary identification of physicochemical properties of extracted soluble dietary fiber of *Lentinus edodes.*

Number	Name of experiment	Experimental phenom	Outcomes
1	Molish reaction	A purple circle is created between the two pages	Presence of saccharides
2	Iodine reaction	Does not show blue	No starch present
3	The ninhydrin reaction	No blue-violet color	No amino acids are present
4	Sulfuric acid-carbazole reaction	Purplish	Presence of glucuronic acid

### 3.2 Molecular weight distribution of soluble dietary fiber in *Lentinus edodes*

**Figure 2:**
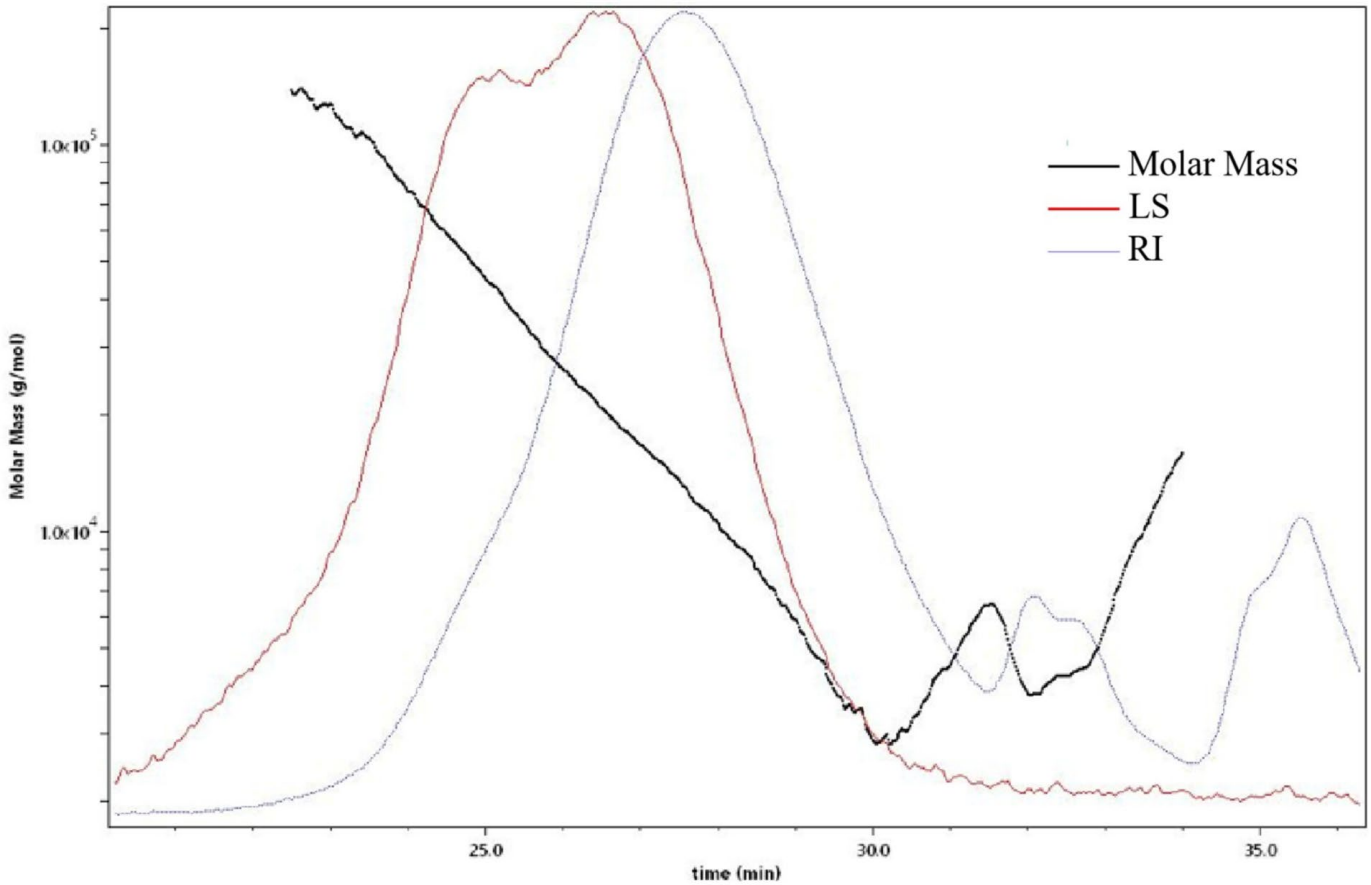
Molecular weight analysis plot of soluble dietary fiber in *Lentinus edodes*, the red line represents the multi-angle laser light scattering signal (i.e., LS, in V), the intensity of scattered light is proportional to the molecular size and molecular weight of the substance; the blue line represents the difference signal (i.e., RI, in RIU), the black line is the molecular weight fitted by the two signals.

The molecular weight distribution of the soluble dietary fiber, In the molecular weight analysis chart (Figure 2), the red line represents the multi-angle laser light scattering signal (i.e., LS, in V), and the intensity of scattered light is proportional to the molecular size and molecular weight of the substance; the blue line represents the difference signal (i.e., RI, in RIU). The response value depends on the change of the outflow refractive index after the column and is related to the type, concentration and molecular weight; the black line is the molecular weight fitted by the two signals. Where the inorganic salt phase around 37 min is the solvent peak of the mobile phase. The molecular weight distribution is shown in Table 4. After analysis, the average molecular weight of soluble dietary fiber of *Lentinus edodes* was 8.356 kDa, the peak molecular weight Mp is 13.124 kDa, the weight Mw is 17.019 kDa, and Z Mz is 36.781 kDa.

**Table 4:** ALT, AST, ACP and γ-GT contents of the liver in each group (x ± s, *n* = 6 U/g).

Group	N	F	FL	FM	FH
ALT	0.104 ± 0.005	0.162 ± 0.012a	0.126 ± 0.004Ab	0.118 ± 0.004b	0.116 ± 0.007b
AST	0.344 ± 0.046	0.530 ± 0.041a	0.379 ± 0.015Ab	0.357 ± 0.006b	0.376 ± 0.035b
ACP	311.974 ± 26.001	454.770 ± 30.065a	376.971 ± 30.496Ab	329.995 ± 13.619b	338.101 ± 36.650b
γ-GT	39.860 ± 2.940	31.203 ± 2.767a	38.416 ± 5.451b	38.973 ± 2.370b	40.008 ± 2.388b

a: *p* < 0.01, A: *p* < 0.05, b: *p* < 0.01, B: *p* < 0.05.

### 3.3 Determination of monosaccharide composition of soluble dietary fiber in *Lentinus edodes*

**Figure 3:**
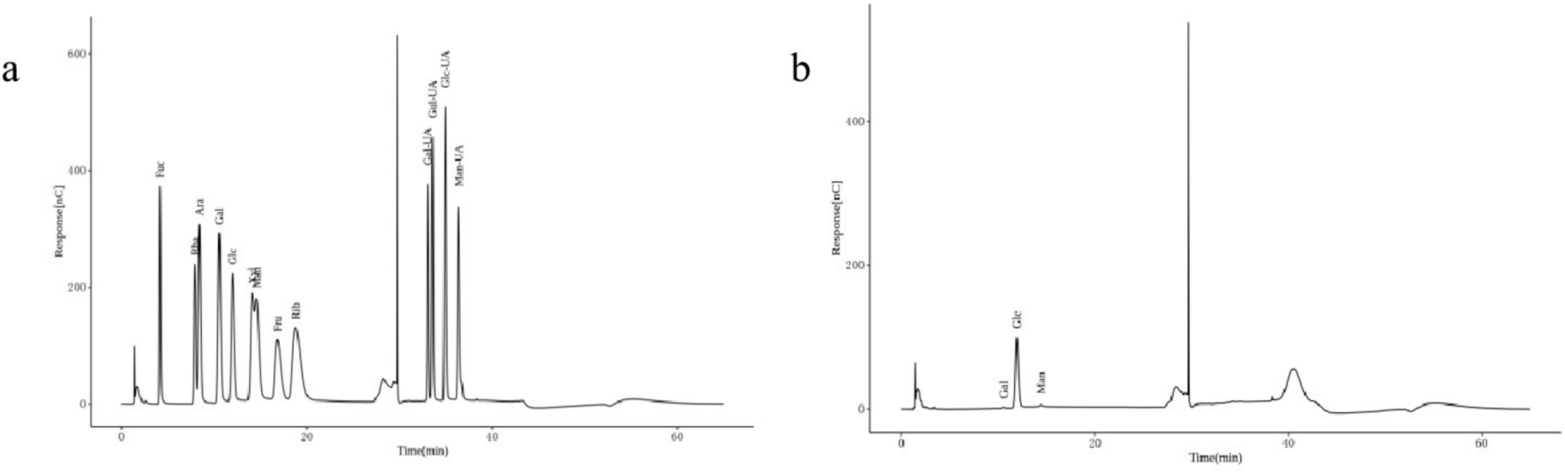
Monosaccharide composition of *Lentinus edodes* soluble dietary fiber. **(A)** Ion chromatograms of the standard sample. **(B)** Ion chromatogram of soluble dietary fiber in *Lentinus edodes.* In panel **(A)**, Fuc is an abbreviation for Fucose, Rha is an abbreviation for Rhamnose, Ara is an abbreviation for Arabinose, Gal is an abbreviation for Galactose, Glc is an abbreviation for Glucose, Xyl is an abbreviation for Xylose, Man is an abbreviation for Mannose, Fru is an abbreviation for Fructose, Rib is an abbreviation for Ribose, Gal-UA is an abbreviation for Galacturonic Acid, Glc-UA is an abbreviation for Glucuronic Acid, Man-UA is an abbreviation for Mannuronic Acid, Gul-UA is an abbreviation for Guluronic Acid.

The chromatographic qualitative was mainly based on the retention time of the target compounds on the analytical column. For elution conditions (gas/liquid), different compounds on the retention ability of analytical column is different, there are many differences, column is mainly through analytical column separation of different compounds, reuse ultraviolet detector, evaporation light detector, differential detector, electrochemical detector, conductance detector, generally can be through the peak time to determine the target compound. Figure 3A shows the ion chromatogram of the standard sample, and Figure 3B shows the ion chromatogram of the extracted mushroom soluble dietary fiber. After comparison, it was found that the monosaccharides in the soluble dietary fiber of the extracted *Lentinus edodes* were mainly galactose, glucose and mannose.

### 3.4 Electron microscopic scanning analysis of soluble dietary fiber in *Lentinus edodes*

**Figure 4:**
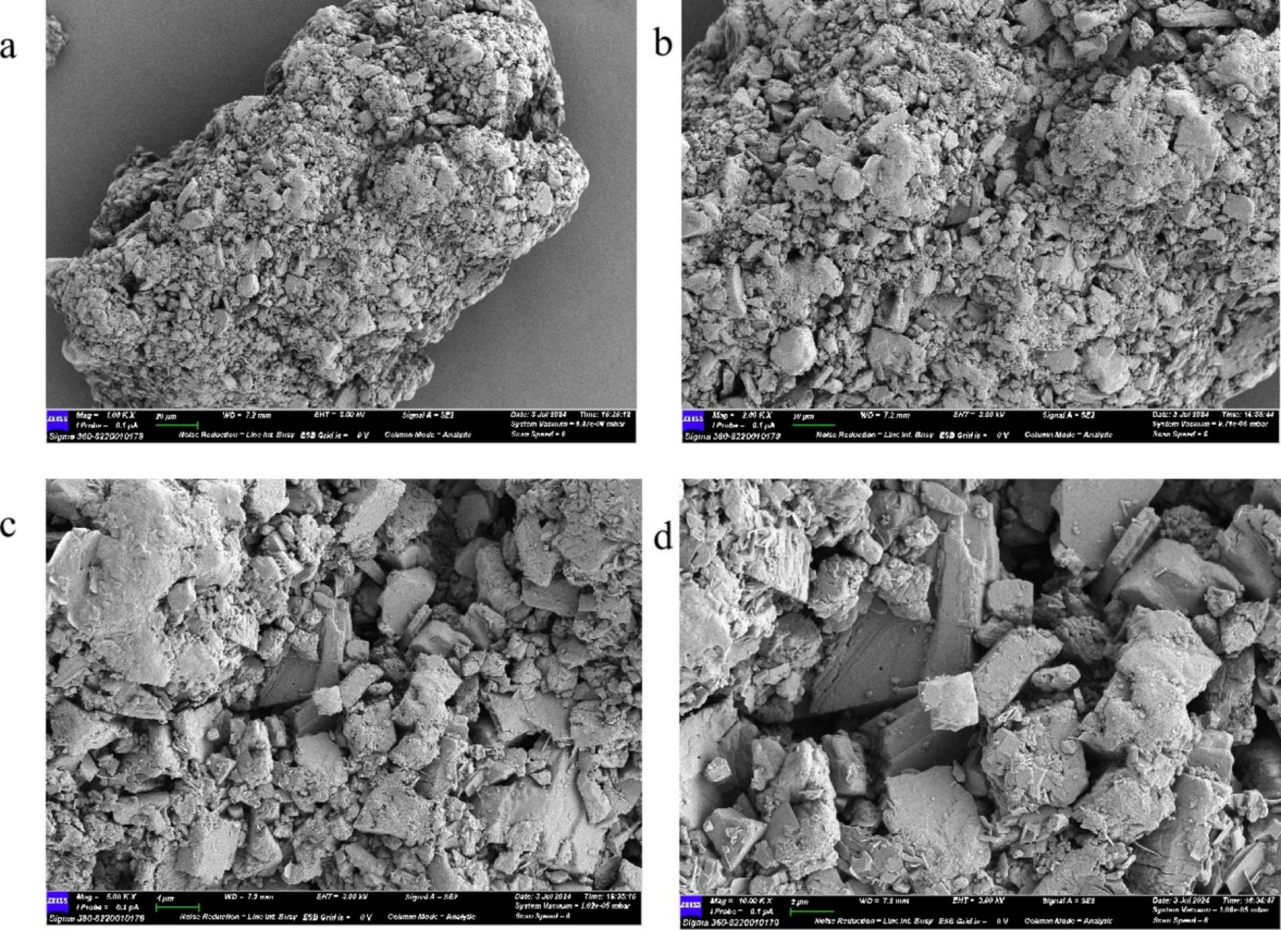
SEM plot of soluble dietary fiber in *Lentinus edodes*. **(A)** 500×, **(B)** 1,000×, **(C)** 2,500×, **(D)** 5,000×.

Using a scanning electron microscope (SEM) on the surface microstructure of mushroom soluble dietary fiber, SEM photos of different magniations of mushroom soluble dietary fiber are shown in Figure 4, at 500× and 1,000×, the soluble dietary fiber of *Lentinus edodes* showed an irregular network shape (Figures 4A,B), higher power with a clearer display as shown in panels Figures 4C,D, the sample surface is uneven, an irregular geometric shape, with a distinct fibrous structure, tightly connected, with a small gap.

### 3.5 Infrared spectroscopy of soluble dietary fiber in *Lentinus edodes*

**Figure 5:**
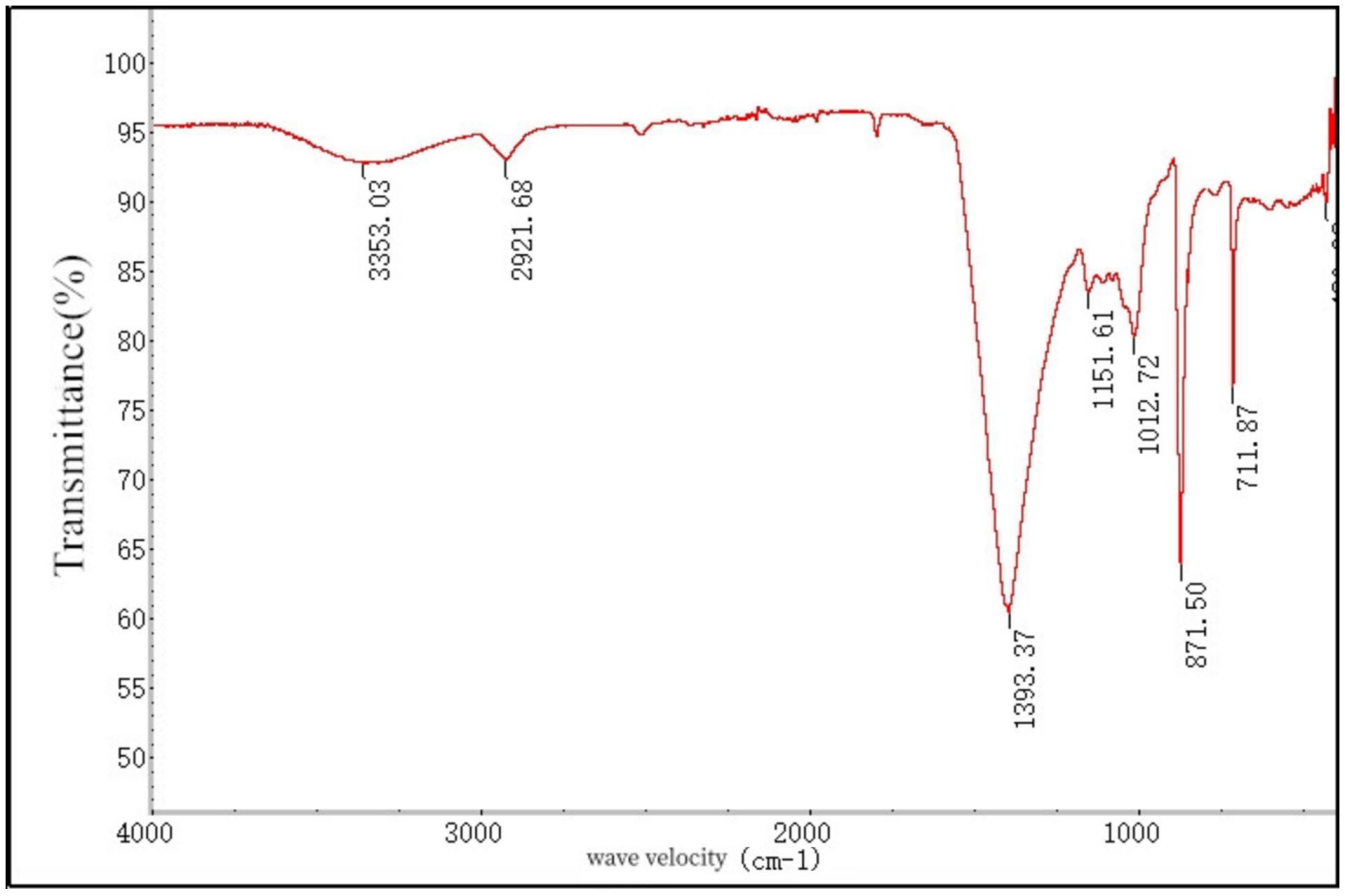
Ired spectrogram of soluble dietary fiber in *Lentinus edodes.*

**Figure 6:**
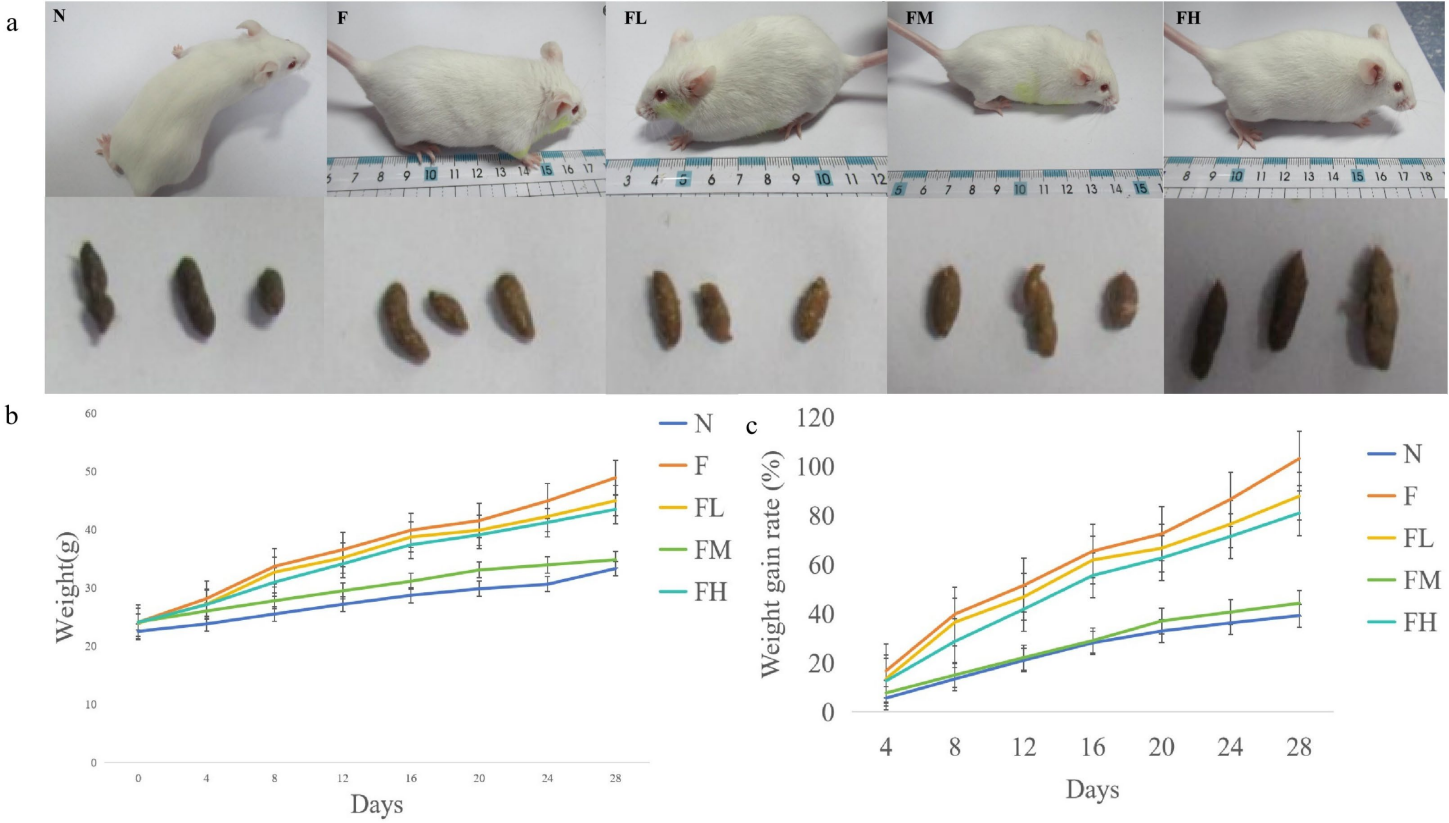
General state of the mice in each laboratory. **(A)** Hair color and feces of mice in each group, **(B)** trend chart of mouse body weight change, **(C)** body weight growth rate chart.

The soluble dietary fiber of *Lentinus edodes* and potassium bromide were pressed into bromide and placed into the Fourier infrared spectrophotometer to determine the infrared absorption spectroscopy at 400–4,000 cm^−1^ (Figure 5). The scan spectrum is shown in Figure 6. According to the atlas, the characteristic peak frequency area at 3,353.03 cm^−1^ in the range of 4,000–650 cm^−1^ is the absorption peak generated by the stretching vibration of sugar intra-molecular or intermolecular O–H, At 2,921.68 cm^−1^ is the absorption peak generated by the C–H expansion vibration, at 1,393.37 cm^−1^ is the C=O expansion vibration peak, 1,151.61 cm^−1^ for C–O–C expansion vibration, 1,012.72 cm^−1^ is the telescopic vibration of the C–O, 711.87 cm^−1^ for the absorption peak generated by the β-substitution, Show that the presence of the β-glycosidic bond in the sample. This infrared map verified the presence of alcoic acid in the soluble dietary fiber of *Lentinus edodes*, the monosaccharides are mainly connected by β-glycosidic bonds.

### 3.6 General state of mice

Before the start of the experiment, the fur of each group of mice was white, smooth, and shiny, the skin was light pink, and the reaction was sensitive. The feces were oval shaped and moderately moist and soft before the experiment. As the experiment progressed, the fur of Group F mice gradually became disheveled, withered, yellow and dull, their skin appeared slightly dull and pale, their bodies were obese, and their feces were wet and oily. The fur of mice in the FL group, FM group, and FH group, which added soluble dietary fiber from *Lentinus edodes*, showed varying degrees of disorder, with the disorder degree being FH > FL > FM, but none of them lost their luster. Striped fibers were clearly visible in the feces, and the feces were dry and hard, with a lower water content than the normal group. The body size of mice in each group was F > FL > FH > FM > =N (Figure 6A).

Weigh the mice at designated locations every 4 days and record their weight changes. The trend of weight changes in each group of mice is shown in Figure 6B. After adaptive feeding, there was no significant difference in the initial weight of mice in each group (day 0). As the experimental days increased, the weight of mice in group F rapidly increased. On day 28, the weight reached 48.87 ± 3.08 g, which was significantly different from group N and FM (*p* < 0.01). On the 4th, 8th, 12th, 16th, 20th, 24th, and 28th days of the experiment, compared with the beginning of the experiment, the weight growth rate is shown in Figure 6C. At the end of the experiment, compared with the 0th day, the weight growth rate of each group is F > FL > FH > FM > N, which is consistent with the observed body size. There is a significant difference between the F group and the N and FM groups (*p* < 0.01), and there is also a significant difference between the FL and FH groups and the N and FM groups (*p* < 0.01). Indicating that soluble dietary fiber from *Lentinus edodes* can to some extent slow down weight gain caused by high-fat diets.

### 3.7 Effects of soluble dietary fiber from *Lentinus edodes* on liver coefficient, spleen coefficient, and thymus coefficient in high-fat diet mice

**Figure 7:**
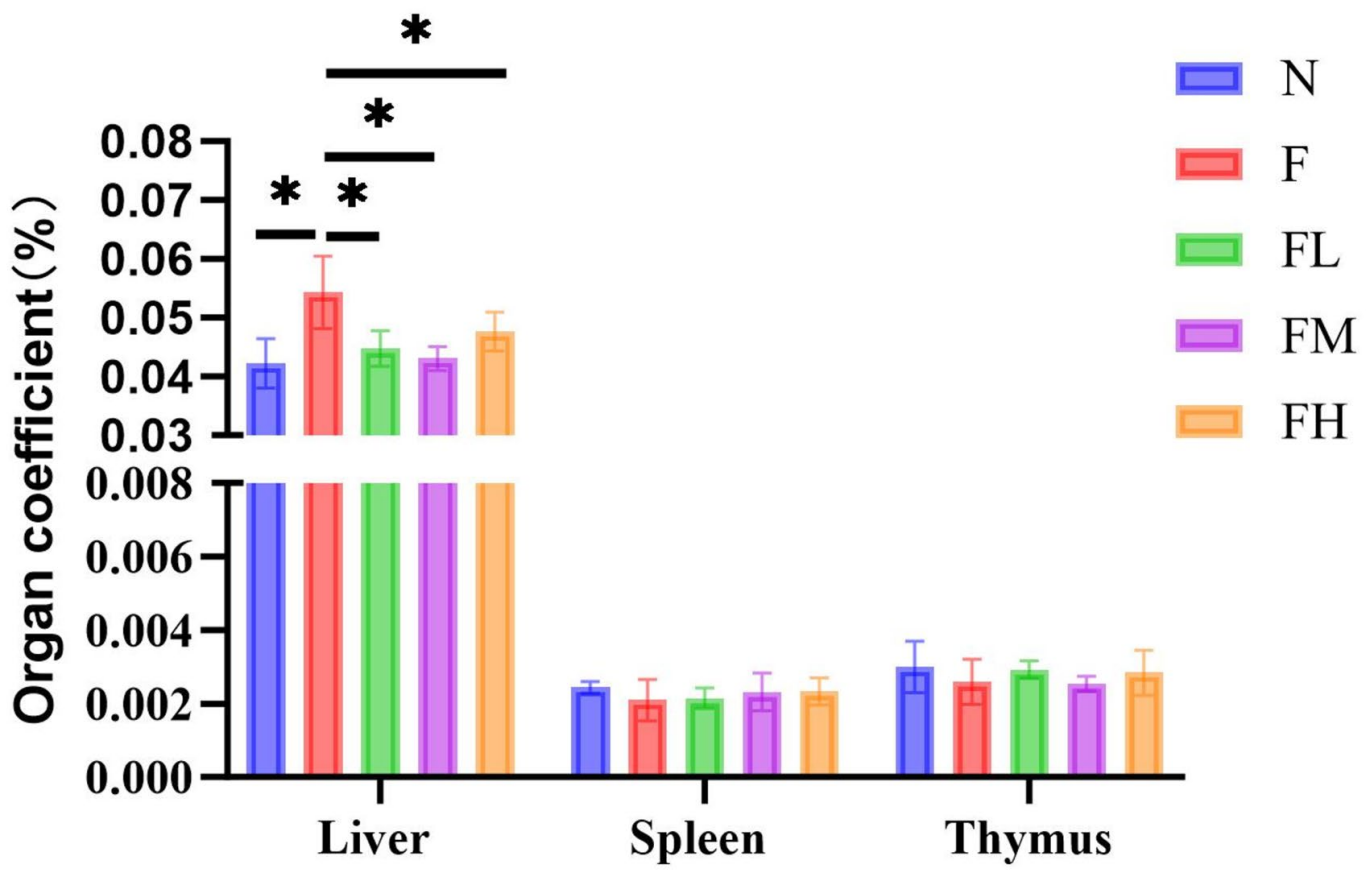
Liver coefficient, spleen coefficient, and thymus coefficient of the mice in each.

Organ coefficient, also known as organ to body ratio, is the ratio of the weight of a certain organ in an experimental animal to its body weight. The ratio of various organs to body weight is relatively constant under normal circumstances. When organs undergo pathological changes, the weight of the damaged organs changes, and the organ coefficient also changes accordingly. From Figure 7, it can be seen that there is no significant difference in the spleen coefficient and thymus coefficient among the experimental groups, indicating that there are no abnormalities in the immune organs spleen and thymus. However, there is a significant difference in the liver coefficient between group F and group N (*p* < 0.01), indicating that the liver of mice fed a high-fat diet for a long time has lipid accumulation, which may have caused lesions. Although the liver coefficients of the FL group, FM group, and FH group slightly increased, there was no significant difference compared to the N group, indicating that there were no organic changes in the liver tissue.

### 3.8 The effect of soluble dietary fiber from *Lentinus edodes* on the morphology of liver tissue in high-fat diet mice

#### 3.8.1 Liver histomorphology of mice in each group

**Figure 8:**
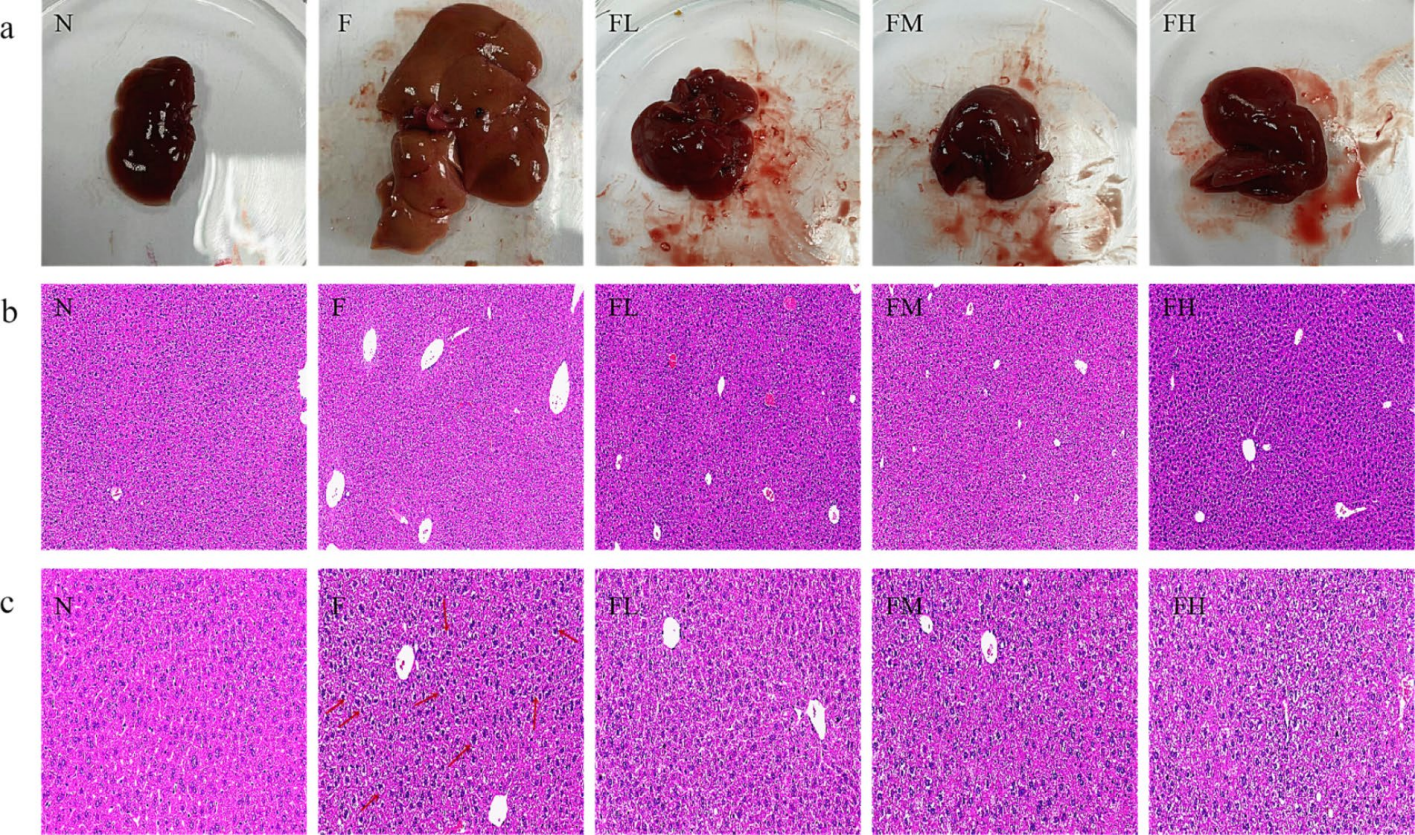
The effect of soluble dietary fiber from *Lentinus edodes* on the morphology of liver tissue in high-fat diet mice. **(A)** Representative pictures of the mouse liver in each group, **(B)** H&E staining of the liver of each mouse group (10.0×), **(C)** H&E staining of the liver of each mouse group (50.0×).

Dissect experimental mice in each group, observe and take photos of their livers (Figure 8A). The livers of mice in group N are reddish brown with a glossy texture, soft and brittle, while those in group F are enlarged, pale in color, rough in texture, and exhibit obvious characteristics of fatty liver. The liver of the FL group, FM group, and FH group appeared bright red with no hypertrophy, and had a soft and crisp texture. The phenotype observation showed no significant difference compared to the N group.

#### 3.8.2 H&E staining of liver in each group of mice

In the evaluation of balloon like changes in NASH-CRN score, if the size of liver cells is similar to that of normal liver cells, the shape is round, and the cytoplasm has pale network, it can be judged as mild fatty liver (20). After the experiment, the liver tissues of mice in each group were embedded and stained with H&E. The experimental results are shown in Figure 8B. The liver cells of mice in Group N were arranged in a regular and orderly manner, with clear cell boundaries and round nuclei. The structure of the liver tissue in Group F showed significant pathological changes, with the disappearance of the liver lobule structure and large round vacuoles (fat droplets) inside the liver cells. Although no pathological changes occurred in the liver tissues of Group FL, Group FM, and Group FH, a small number of small round vacuoles were observed. As shown in Figure 8C, compared with other experimental groups, the liver lobule structure in group F was disordered, the hepatic cord was crowded, the volume of hepatocytes was significantly increased, the size was different, the cytoplasm of hepatocytes was loosely arranged, some hepatocytes were spherical, the cytoplasm was transparent and vacuolated, and there was balloon like change. The above results indicate that soluble dietary fiber from *Lentinus edodes* can significantly delay liver cell damage in mice fed a high-fat diet.

### 3.9 The impact of soluble dietary fiber from *Lentinus edodes* on liver injury factors in mice fed a high-fat diet

#### 3.9.1 Effects of soluble dietary fiber from *Lentinus edodes* on liver ALT and AST levels in mice fed a high-fat diet

ALT and AST are important indicators to reflect whether there is liver injury and the severity of liver injury. ALT was mainly distributed in the cytoplasm of hepatocytes, and the increase of ALT reflected the damage of hepatocyte membrane; AST was mainly distributed in the cytoplasm and mitochondria of hepatocytes, and its increase suggested that hepatocytes were damaged to the level of organelles. The contents of ALT and AST of mice in each group are shown in Table 5. Compared with group n, the serum ALT and AST levels of mice in group F were significantly increased (*p* < 0.01), the serum ALT and AST levels of mice in group FL were between group n and group F (*p* < 0.01 or *p* < 0.05), and there was no difference between group FM and group FH and group N.

**Table 5:** Effect of soluble dietary fiber of *Lentinus edodes* on hepatic lipid metabolism level in mice with high-fat diet (x ± s, *n* = 6 mmol/L).

Group	N	F	FL	FM	FH
TG	3.027 ± 0.277	4.770 ± 0.290a	4.780 ± 0.470a	3.947 ± 0.245AB	4.886 ± 0.491a
TC	1.435 ± 0.082	2.558 ± 0.225a	1.828 ± 0.059Ab	1.445 ± 0.270b	1.800 ± 0.090ab
FFA	368.300 ± 30.312	862.588 ± 24.328a	785.946 ± 139.682a	635.185 ± 29.728ab	570.997 ± 66.901ab

a: *p* < 0.01, A: *p* < 0.05, b: *p* < 0.01, B: *p* < 0.05.

#### 3.9.2 Effects of *Lentinus edodes* soluble dietary fiber on ACP and γ-GT in liver of mice fed with high fat diet

ACP and γ-GT are important indicators to determine whether liver fat accumulation and liver tissue lesions. As shown in Table 4, ACP level in group F increased significantly compared with group N (*p* < 0.01 or *p* < 0.05), and γ-GT level decreased significantly, suggesting that fat accumulation and poor excretion in the liver due to long-term high-fat diet. No difference in the levels of ACP and γ-GT in the FL, FM and FH groups was compared with the group N.

In conclusion, a certain amount of *Lentinus edodes* soluble dietary fiber can significantly reduce the levels of alt, AST and ACP in the liver and restore the level of γ-GT, indicating that *Lentinus edodes* soluble dietary fiber can improve the liver injury caused by high-fat diet.

### 3.10 Effect of *Lentinus edodes* soluble dietary fiber on liver lipid metabolism in high fat diet mice

#### 3.10.1 Effect of *Lentinus edodes* soluble dietary fiber on the level of liver lipid transporter in mice fed with high-fat diet

**Figure 9:**
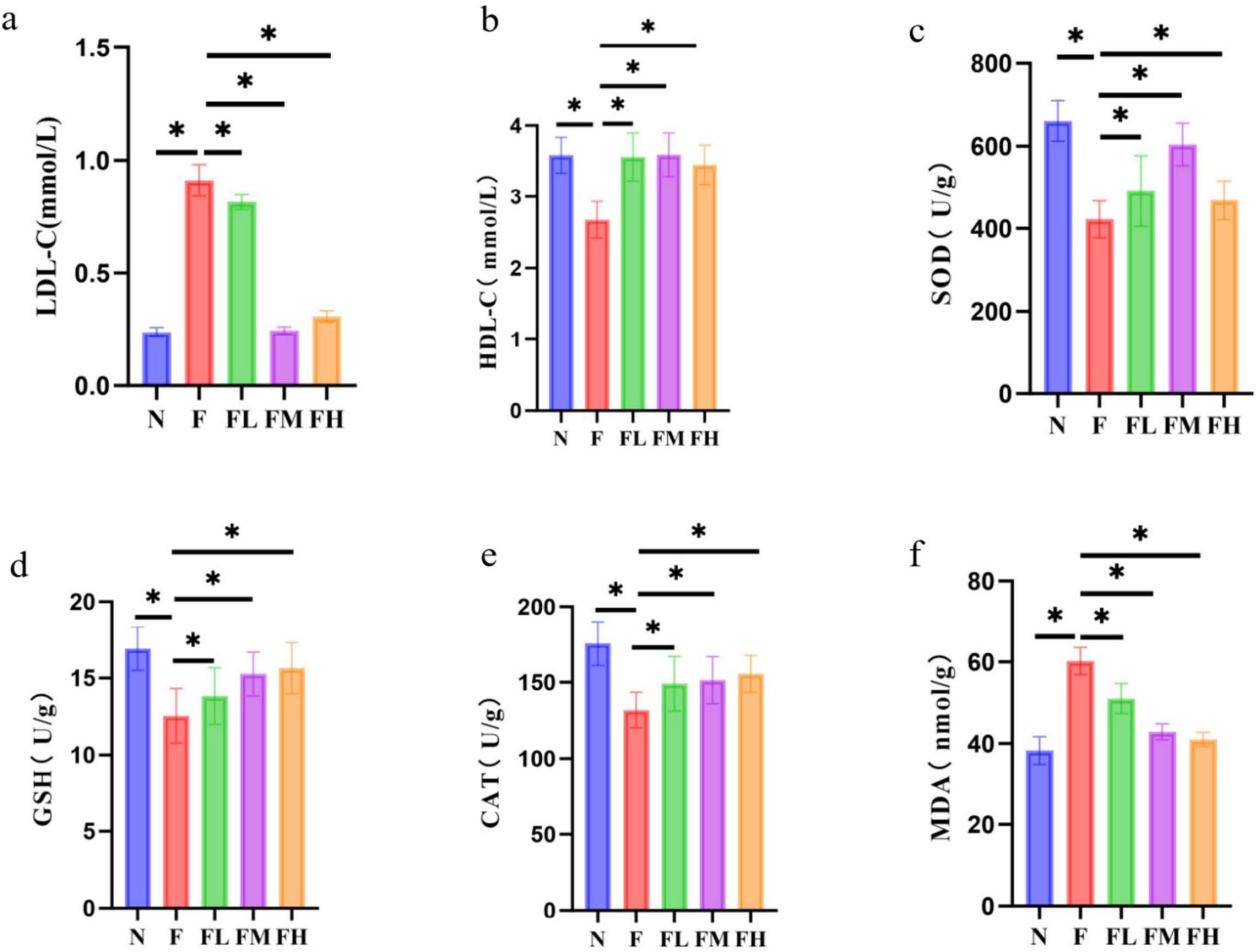
Effect of *Lentinus edodes* soluble dietary fiber on lipid transporter levels and oxidative stress levels in mice fed a high-fat diet. **(A)** Serum levels of LDL-C in each mouse group, **(B)** serum levels of HDL-C in each mouse group, **(C)** the level of the SOD in the liver, **(D)** the level of the GSH in the liver, **(E)** the level of the CAT in the liver, **(F)** the level of the MDA in the liver.

The levels of serum LDL-C and HDL-C can reflect the cholesterol transport in the vascular wall. LDL-C enters the vascular wall through the vascular endothelium, and the LDL-C retained in the subendothelium is modified into oxidized LDL-C (ox LDL-C). Macrophages phagocytize ox LDL-C and form foam cells. The latter increases and fuses, forming the lipid core of atherosclerotic plaque. A large number of studies have shown that atherosclerosis is a chronic inflammatory disease, and LDL-C is likely to be the basic element of the initiation and maintenance of this chronic inflammatory reaction. HDL-C can transport cholesterol in the vascular wall to the liver for catabolism (i.e., reverse cholesterol transport), reduce the deposition of cholesterol in the vascular wall, and play an anti atherosclerotic role, so it is called “good” cholesterol. The levels of serum LDL-C and HDL-C of mice in each laboratory are shown in Figures 9A,B. Compared with group N, the levels of LDL-C in group F and FL increased significantly (*p* < 0.01), and the levels of HDL-C in group F decreased significantly, while the levels of LDL-C and HDL-C in FM and FH groups had no difference compared with group N.

#### 3.10.2 Effect of *Lentinus edodes* soluble dietary fiber on liver lipids in mice fed with high-fat diet

It can be seen from Table 5, compared with group N, the levels of TG, TC and FFA in group F, FL and FH were significantly increased (*p* < 0.01 or *p* < 0.05), while the levels of TG and FFA in group FM were significantly increased (*p* < 0.01 or *p* < 0.05), but the level of TC was not different from that in group n. Compared with group F, the levels of TG, TC and FFA in group FL, FM and FH were decreased (*p* < 0.01 or *p* < 0.05).

In conclusion, a certain amount of *Lentinus edodes* soluble dietary fiber can effectively reduce the level of LDL-C and restore the level of HDL-C trans lipoprotein, and effectively reduce the levels of serum TG, TC and FFA. The results showed that a certain amount of *Lentinus edodes* soluble dietary fiber had lipid-lowering effect on high-fat diet mice.

### 3.11 Effect of *Lentinus edodes* soluble dietary fiber on oxidative stress in mice fed with high fat diet

It can be seen from Figures 9C–F that the high-fat diet led to the decrease of liver SOD, GSH and cat levels compared with group N (*p* < 0.01), the transformation ability of SOD, GSH and cat was weakened, and the MDA level was significantly increased compared with group N (*p* < 0.01), the damage of hepatocyte membrane was aggravated. The levels of SOD, GSH, cat and MDA in the liver of FL group, FM group and FH group added with *Lentinus edodes* soluble dietary fiber were all decreased, and there was no difference between FM group and FH group and N group (*p* > 0.05), indicating that a certain amount of *Lentinus edodes* soluble dietary fiber can effectively improve the oxidative stress caused by high-fat diet.

### 3.12 Effect of *Lentinus edodes* soluble dietary fiber on the liver lipid metabolism in mice fed with high fat diet

**Figure 10:**
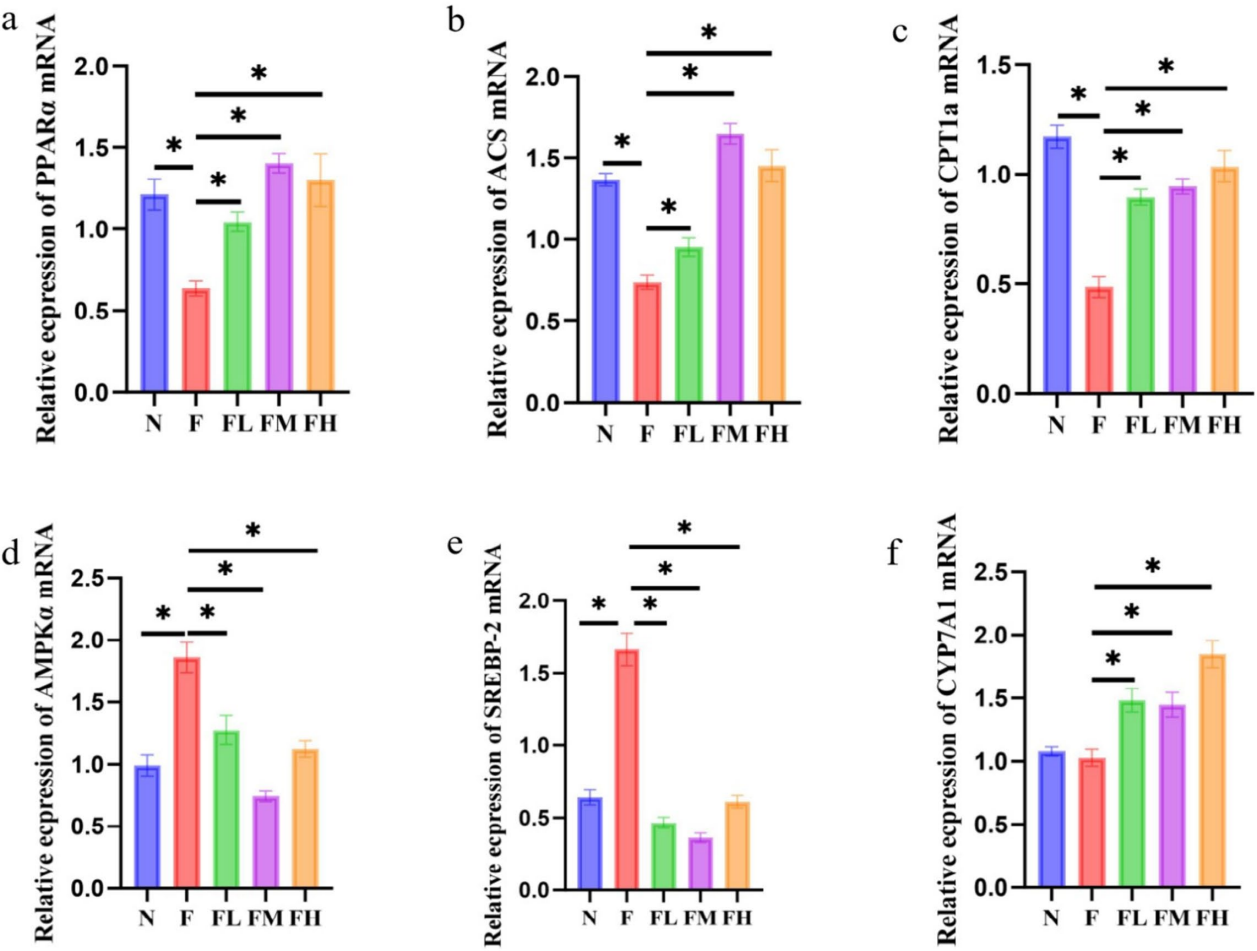
Relative expression of liver-related genes in soluble dietary fiber in high-fat diet. **(A)** PPAR-α/NADPH, **(B)** ACS/NADPH, **(C)** CPT1a/NADPH, **(D)** AMPKα/NADPH, **(E)** SERBP2/NADPH, **(F)** CYP7A1/NADPH.

Liver is an important organ involved in lipid metabolism, in which there are many enzymes and transcription factors related to lipid metabolism. The effect of *Lentinus edodes* soluble dietary fiber on the expression of genes related to liver lipid metabolism in mice fed with high fat diet is shown in Figure 10. The high-fat diet led to a decrease in the expression of PPAR-α, ACS and CPT1a genes in the liver of mice, and an increase in the expression of AMPKα and SREBP-2 genes, with no significant effect on the gene expression of CYP7A1. *Lentinus edodes* soluble dietary fiber intervened in high-fat diet, increased the expression of PPAR-α, ACS, CPT1A and CYP7A1 genes in the liver of mice, and significantly decreased the expression of AMPKα and SREBP-2.

## 4 Discussion

### 4.1 The relationship between the soluble dietary fiber structure of *Lentinus edodes* and its lipid-lowering efficacy

Monosaccharide composition and glycosidic bonds of molecules have important effects on the function of soluble dietary fiber. Studies have shown that the soluble dietary fiber in fungi is mainly composed of galactose, glucose and mannitol in different proportions (21). Studies have also shown that arabinose, xylose, fucose, ribose, rhamnose, etc. are also detected in the soluble dietary fiber in fungi (10, 22, 23). The soluble dietary fiber extracted from *Lentinus edodes* in this study is mainly composed of galactose, glucose and mannitol, which is mainly connected by β-glycosidic bonds. Schulthess and other researchers have shown that glucose and mannitol can be used by anaerobic bacteria in the intestine to metabolize a large amount of acetic acid, which is conducive to reducing the proportion of pathogenic bacteria and maintaining the integrity of the intestinal barrier. Galactose, glucose and mannitol in *Lentinus edodes* soluble dietary fiber have a good regulatory effect on intestinal flora, can affect the secretion of intestinal SCFAs, and maintain the integrity of intestinal barrier. Compared with α-glycosidic bond, β-glycosidic bond is more likely to form a vertical conformation. Studies have shown that dextran linked by β-(1,4) glycosidic bond is more prone to intramolecular aggregation, resulting in reduced solubility, while β-(1,3) bond in glycosidic bond can reduce the polymerization, making dextran in a dissolved state in the body, which is more likely to be used and fermented by intestinal microorganisms (24). It has been reported that the main chain of lipid-lowering polysaccharides from edible fungi (Pleurotus ostreatus and Pleurotus ostreatus) is β-(1,3)-glucan, and contains a certain amount of β-(1,3)-glucose side chain. The glycosidic bond of *Lentinus edodes* soluble dietary fiber is β-glycosidic bond. The related structure may play the physical role of adsorbing lipids or bile salts *in vivo*, regulating intestinal flora and increasing the content of intestinal SCFAs.

The molecular weight (Mw) also plays an important role in the efficacy of soluble dietary fiber. When the molecular weight of soluble dietary fiber is too large, it will be difficult for organisms to absorb and utilize because the molecular weight of the substance is too large to pass through the cell membrane. When the molecular weight of soluble dietary fiber is too low, it will be difficult to form active substances, resulting in the reduction of physiological activity. The weight average molecular weight of soluble dietary fiber from *Lentinus edodes* is 17.019 kDa, and its molecular weight is uniform. Studies have shown that (25–28), the biological activity of polysaccharides is negatively correlated with the relative molecular weight. Some researchers isolated five kinds of Polysaccharides from *Lycium barbarum*, of which IBPA 4 with a molecular weight of 10.2 kDa showed good anti-cancer activity, while ibp-p 8 with a molecular weight of 6.5 × 103 kDa did not show anti-cancer activity. It may be due to the difference in the three-dimensional structure corresponding to the relative size of molecular weight, resulting in the difference in physiological activity.

### 4.2 The soluble dietary fiber of *Lentinus edodes* has a good lipid-lowering effect and liver protection effect

The liver plays an important role in lipid synthesis, metabolism, and transport processes (29). When the balance between energy intake and consumption is broken, the lipid metabolism of the liver is abnormal, and the lipids in the liver cells begin to accumulate (30, 31). According to the data released by the China Center for Disease Control and prevention, the overweight and obesity rates of all age groups in China are increasing rapidly. At present, about 19% of Chinese adolescents aged 6–17 are overweight and obese, while the proportion among adults is more than 50%, higher than the international level (32). A large of studies (33–35) have found that dietary fiber can reduce fat and weight, prevent obesity, improve insulin resistance, reduce the absorption of food fat, inhibit the synthesis of cholesterol in the liver, and reduce the accumulation of fat in the liver. Long term high-fat diet made the weight and liver coefficient of mice in Group f the highest among the five experimental groups. After the experiment, it was found that the isolated liver of mice fed with high-fat diet was significantly white. H&E staining showed that the liver cells were disordered, and the levels of ALT and AST in the liver were significantly increased. Further detection of the lipid level in the liver of mice fed with high-fat diet showed that the levels of FFA, TG and TC in the liver were significantly increased. All of the above indicate that high-fat diet leads to liver damage and lipid metabolism disorder. However, the liver of mice fed with high-fat diet supplemented with *Lentinus edodes* soluble dietary fiber was still bright red, and the levels of alt, AST, FFA, TG and TC in the liver were all corrected. The lipid accumulation in the liver was reduced, and the liver lipid was significantly reduced, which confirmed that *Lentinus edodes* soluble dietary fiber had a better lipid-lowering effect. Through the comprehensive analysis of the general state, weight gain, organ changes, liver injury, lipid metabolism and oxidative stress reaction of mice fed with high-fat diet supplemented with different doses of *Lentinus edodes* soluble dietary fiber, it was found that daily supplementation of 300 mg/kg *Lentinus edodes* soluble dietary fiber could achieve the effect of lipid-lowering and liver protection.

Liver lipid metabolism disorder is usually manifested as dyslipidemia (36). The levels of TG and TC in mice fed with high-fat diet were significantly abnormal, indicating that high-fat diet led to dyslipidemia in mice. TG is the energy source of muscle and adipose tissue and one of the most critical plasma lipids in clinic. Plasma TG comes from food and liver synthesis (37–39). Fatty acids are absorbed by cell fatty acid transporters (40), and TG is synthesized by diacylglycerol o-acyltransferase and other steps (41). Because the size of chylomicrons (CM) rich in TG limits the penetration of the arterial wall, the increase of TG indirectly promotes atherosclerosis mechanisms such as inflammation, thrombosis and abnormal cell proliferation (42). Cholesterol in the blood is mainly transported by apolipoprotein binding to lipoprotein to form corresponding lipoprotein cholesterol. Low density lipoprotein plays an important role in cholesterol transport (43). After cholesterol is synthesized in the liver, it combines with LDL apolipoprotein to produce LDL-C into the blood. LDL-C is transported to the lysosome through receptor-mediated endocytosis (44). LDL-C enters the inner wall of blood vessels. After the vascular endothelial layer is modified and oxidized to oxidized low density lipoprotein cholesterol, it is engulfed by macrophages to form foam cells (45). Foam cells continue to increase and fuse, forming the lipid core of atherosclerotic plaque. The liver is the largest metabolic organ in the body. GSH is an important antioxidant in the body and can eliminate ROS (46). Therefore, the change of GSH content can be regarded as a sign of oxidative stress imbalance. When the organs are in a state of overload metabolism, a large number of ROS will be produced, accompanied by the decrease of GSH content in the liver. SOD, GSH and cat play an important biological role in organisms (47). They can transform reactive oxygen species into harmless substances and protect cells from oxidative damage through their cooperative effect. At the same time, they can also be used as indicators of the degree of oxidative stress in organisms, reflecting the health status of organisms (48). The content of MDA is an indicator of cell membrane peroxidation. The levels of GSH, SOD and cat in the liver of mice fed with high-fat diet were significantly decreased, while the level of MDA increased, while the levels of GSH, SOD, cat and MDA in the experimental group supplemented with medium and high doses of *Lentinus edodes* soluble dietary fiber were all corrected.

### 4.3 The soluble dietary fiber of *Lentinus edodes* can affect the expression of genes in hepatic lipid metabolism to reduce lipid

**Figure 11:**
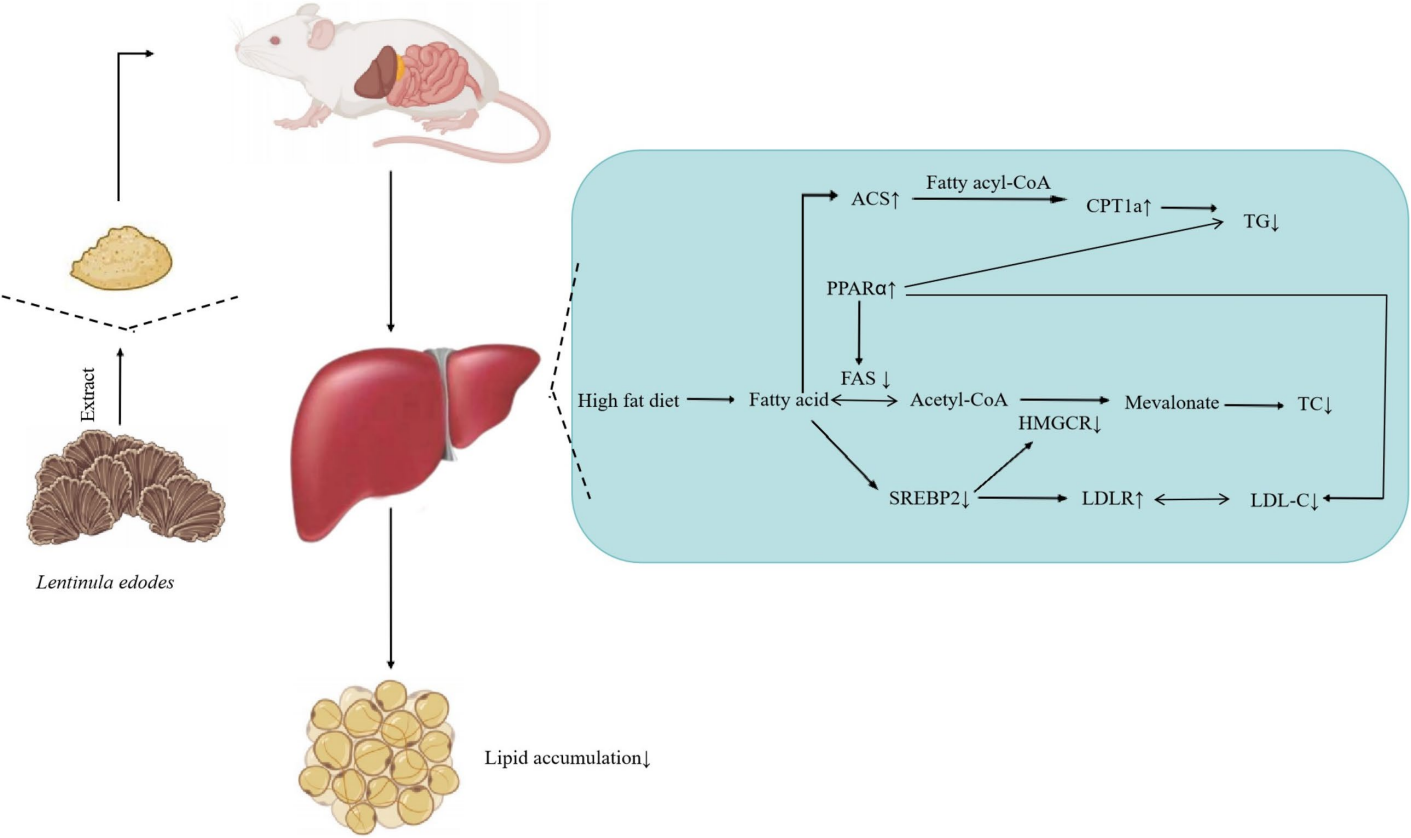
Effect of soluble dietary fiber on hepatic lipid, *Lentinus edodes* soluble dietary fiber can reduce the liver TG level by up regulating the gene expression of ACS and PPARα, reduce the liver TC level by down regulating the gene expression of Fas and SREBP2, and reduce the LDL-C level by down regulating the gene expression of SREBP2.

Liver is an important organ involved in lipid metabolism, in which there are many enzymes and transcription factors related to lipid metabolism. Peroxisome proliferator activated receptor α (PPARα) is a ligand activated nuclear transcription factor in the nuclear receptor superfamily (49). It can affect lipid metabolism by regulating the expression of acyl CoA oxidase (acox) and cytochrome P4504A (CYP4A), and also plays an important role in blood glucose homeostasis and insulin resistance. Acyl CoA synthase (ACS) is a large and diverse enzyme family, which can be divided into different types according to the length of fatty acid substrate chain (50). In the process of fatty acid metabolism, ACS catalyzes the occurrence of initial reaction to synthesize fatty acid CoA, thus activating fatty acid oxidation in cells. Carnitine palmityl transferase 1α (CPT1α) is a member of the carnitine palmityl transferase-1 (CPT1) family. The enzyme family is located on the outer membrane of mitochondria. It catalyzes the synthesis of fatty carnitine from fatty CoA and carnitine, and mediates the transfer of long-chain fatty CoA into the mitochondria for oxidation. The entry of fatty acid CoA into mitochondria is the rate limiting step of fatty acid β-oxidation. Therefore, CPT1*α* is the rate limiting enzyme of fatty acid β-oxidation. High fat diet reduced the mRNA expression of PPARα, ACS and CPT1α genes in the liver of mice, and different doses of *Lentinus edodes* soluble dietary fiber intervention could reduce this change (51). AMPKα, the catalytic subunit of AMPK known as “energy sensors,” plays a key role in maintaining the energy balance of AMPK and the dynamic balance of lipid metabolism. SREBP-2 is a member of the sterol regulatory element-binding protein family that mainly regulates gene expression related to cholesterol synthesis and uptake (52), therefore, downregulation of hepatic SBREP-2 expression reduces plasma cholesterol synthesis. Different doses of polysaccharide intervention significantly downregulated the mRNA expression of AMPKα and SREBP-2 genes, indicating that the mushroom soluble dietary fiber has a strong ability to inhibit *de novo* cholesterol synthesis (53). Liver X receptor α (LXRα) is the main nuclear receptor regulating cholesterol metabolism. It regulates the expression of cholesterol metabolism-related genes, such as SREBP-1c, FAS, CYP7A1, Acetyl-CoA carboxylase (ACC), Stearyl-CoA desaturase 1 (SCD 1), and promotes the liver lipid generation (54). Among them, CYP7A1 is the rate-limiting enzyme that catalyzes the synthesis of bile acids by cholesterol from the classical pathway (55). The cholesterol in the body is removed by the formation of bile acids under the action of CYP7A1, which plays an important role in maintaining the dynamic balance of cholesterol and bile acid levels (56, 57). The *Lentinus edodes* soluble dietary fiber intervention significantly upregulated CYP7A1 gene expression, indicating it could effectively promote the conversion of cholesterol into bile acid. The above results show that the soluble dietary fiber of mushroom can promote the conversion of cholesterol into bile acid, and effectively reduce the total cholesterol content of the body (Figure 11).

In summary, in the case of high-fat diet, supplementation of 300 mg/kg of *Lentinus edodes* soluble dietary fiber daily, which can achieve the effect of lipid-lowering and liver protection. However, it is not clear whether the *Lentinus edodes* soluble dietary fiber has an impact on the intestinal microecology, and whether the *Lentinus edodes* soluble dietary fiber further affects the lipid metabolism of the liver by affecting the “gut liver” axis, which will be the focus of our follow-up research.

## Data Availability

The original contributions presented in the study are included in the article/supplementary material, further inquiries can be directed to the corresponding authors.
